# Optimized time dependent exfoliation of graphite for fabrication of Graphene/GO/GrO nanocomposite based pseudo-supercapacitor

**DOI:** 10.1038/s41598-023-41309-9

**Published:** 2023-08-30

**Authors:** Sana Zainab, Sajal Fraz, Saif Ullah Awan, Danish Hussain, Syed Rizwan, Waqar Mehmood

**Affiliations:** 1 Department of Electrical Engineering, NUST College of Electrical and Mechanical Engineering, National University of Sciences and Technology (NUST), Islamabad, 54000 Pakistan; 2 Department of Mechatronics Engineering, NUST College of Electrical and Mechanical Engineering, National University of Sciences and Technology (NUST), Islamabad, 54000 Pakistan; 3 Physics Characterization and Simulations Lab, School of Natural Sciences (SNS), National University of Sciences and Technology (NUST), Islamabad, 54000 Pakistan; 4 Material Synthesis & Characterizations (MSC) Laboratory, Department of Physics, Fatima Jinnah Women University (FJWU), The Mall, Rawalpindi, 46000 Pakistan

**Keywords:** Energy science and technology, Engineering, Materials science, Nanoscience and technology, Physics

## Abstract

High capacitance devices (Supercapacitors) fabricated using two-dimensional materials such as Graphene and its composites are attracting great attention of the research community, recently. Synthesis of 2D materials and their composites with high quality is desirable for the fabrication of 2D materials-based supercapacitors. Ultrasonic Assisted Liquid Phase Exfoliation (UALPE) is one of the widely used techniques for the synthesis of graphene. In this article, we report the effect of variation in sonication time on the exfoliation of graphite powder to extract a sample with optimal properties well suited for supercapacitors applications. Three different graphite powders (hereafter termed as sample A, sample B, and sample C) were sonicated for duration of 24 h, 48 h and 72 h at 60 °C. The exfoliation of graphite powder into graphene, GO and GrO was studied using XRD and RAMAN. AFM and SEM were further used to examine the layered structure of the synthesized nanocomposite. UV–visible spectroscopy and cyclic voltammetery were used to measure the band gaps, and capacitive behavior of the samples. Sample B exhibited a remarkable specific capacitance of 534.53 F/g with charge specific capacity of 530.1 C/g at 1 A/g and energy density of 66 kW/kg. Power density varied 0.75 kWh/kg to 7.5 kWh/kg for a variation in current density from 1 to 10 A/g. Sample B showed capacitive retention of 94%, the lowest impedance and highest degree of exfoliation and conductivity as compared to the other two samples.

## Introduction

Energy storage devices have always been a blooming research area due to continuous rise in energy demand. Different type of energy storage devices has been realized which store energy chemically, electrochemically, mechanically, kinetically, magnetically and thermally etc. For example, fuel cells, batteries, fly wheels, hydro pumps and super magnets etc. Due to constant rise in fossil fuel prices (fear of depletion), and rise in eco-friendly energy has increased demand of energy storage devices. Electrochemical energy storage devices use chemical energy in their active material to generate electrical energy through oxidation reduction reactions, efficiently and cost effectively^1^. These devices exhibit high energy density or high power density well suitable for current and future portable electronics. However, now a days, achievement of high energy density and power density with same material is desirable^2^. Super-capacitors exhibit high specific capacitance and high energy density making them a suitable candidate for energy storage applications.

Two Dimensional materials (2D materials) have thickness in nanoscale and they exhibit superior electronic and mechanical properties such as electron mobility, conductivity and mechanical strength. Numerous 2D materials are candidates for energy storage applications such as Graphene, Transition metal dichalcogenides (TMDCs), Transition metal carbide or nitrides (MXenes) and hexagonal boron nitride (h-BN) etc. Mostly 2D materials are exfoliated from their 3D precursor, for example, graphene is mechanically exfoliated from Graphite. In graphite each carbon atom is covalently bonded with three other carbon atoms and each carbon atom is sp^3^ hybridized. These carbon atoms are arranged in hexagonal honeycomb layers with weak Van der Waal's forces between the layers. Graphene can be exfoliated from graphite due to this weak interlayer bonding. Graphene shows extraordinary properties like flexibility, unbeatable thermal conductivity, and electronic behavior. Graphene monolayer is a zero-bandgap material with a sheet of *sp*^*2*^ hybridized carbon atoms. Due to its zero bandgap, its use in semiconducting devices is limited.

Monolayer graphene was first theoretically explored by Wallace in 1947^3^. Andre Geim and Kostya Novoselov used micromechanical cleavage technique to extract graphene from graphite^4^. Micromechanical cleavage or scotch tape method is a top down approach in which bulk material is used and exfoliated to nanoscale. It gives monolayer of graphene but it is not a scalable process and is very tedious with impurities attached with graphene sheet. Different bottom up techniques are used for graphene fabrication such as chemical vapor deposition (CVD), Plasma enhanced chemical vapor deposition (PECVD), Liquid phase exfoliation (LPE) and Ultrasonic Assisted Liquid Phase Exfoliation (UALPE). Chemical vapor deposition(CVD)used for deposition of graphene on metallic substrates and its transfer on different substrates like SiC are introduced and optimized^5,6^. The maximum size of single crystal grown on dielectric substrate by CVD is in range of a micron^7^. Plasma enhanced chemical vapor deposition(PECVD)overcomes disadvantages of CVD by growing graphene at lower temperature and less time while compromising the quality of the domain size^8,9^. 3D graphene films are grown on nanoporous anodic alumina by using PECVD^10^. Liquid phase exfoliation (LPE) is a simple, cheap and scalable method for exfoliation of graphene. In this method there is no temperature constraint and graphene is obtained in form of suspension and can be used for fabrication of electronic devices. However, some solvents may require large sonication time for exfoliation and graphene quantity in dispersion is very low. Recently, graphene nanoplatelets are exfoliated from graphite in a relatively less sonication time of 3 h using graphite oxide as dispersing agent^11^. Graphene like material is obtained by intermittent shearing of commercial graphite powder in DI water using Ultra Plus Konzentrat surfactant^12^. Use of simple dispersing agents is very crucial for commercializing graphene synthesis. In Ultrasonic Assisted Liquid Phase Exfoliation (UALPE), powder is dispersed in a solvent and ultrasonic sound waves are passed through the dispersion to produce graphene dispersions.

In 1958 the famous Hummer’s modified method was introduced. Strong chemicals used in this method change graphite to graphite oxide (GrO) and introduce functional groups to the lateral plane like hydroxyl (OH) and epoxide groups^13^. Due to these functional groups, hydrophilicity is introduced in GrO. When GrO is exfoliated it results in formation of graphene oxide (GO). GrO shows promising prospects for mass scale production of graphene^14^. Oxygen intercalation during exfoliation of graphite powder results in high inter layer spacing and also helps in easy exfoliation of GrO^15^. GO shows better capacitive behavior and properties than graphite due to its lower bandgap, increased surface area which results in more accessible sites for redox reactions^16^. Oxygen provides inter layer distance creating a facile charge transfer network which increases interlayer distance and enhances surface area which facilitates remarkable charge transfer and additional pseudocapacitance^17^. The oxygen functional groups are responsible for the change of carbon atoms hybridization from *sp*^*2*^ to *sp*^*3*^^18^. To increase capacitive behavior of graphene, pseudocapacitance property is compulsory which is induced by addition of oxygen functional group in graphene. GO has been synthesized using electrochemical exfoliation process in which two graphite rods were immersed in SDS solution and exfoliated for 24 h by applying different voltages^19,20^.

Mitra et al. reported graphite as an electrode for supercapacitors with capacitance values ranging from 0.74 to 0.98 mF/cm^2^^21^. Graphene is a promising candidate when used as electrode in supercapacitors due to high specific capacitance and high energy density in different electrolytes. Chenguang et al. reported specific capacitance of 154.1 F/g and specific energy density of 85.6Wh/kg using high surface area graphene^22^. To improve the specific capacitance of graphene, surfactant stabilization was used which gave specific capacitance upto 194 F/g^23^. Santhakumar et al. reported a graphene based supercapacitor with specific capacitance of 195 F/g and energy density of 83.4 Wh/kg^24^. Ideally, specific capacitance for a monolayer of sp^2^ bonded carbon atoms (monolayer graphene) can reach upto ~ 550 F/g which sets an upper limit on graphene’s specific capacitance^25^. Laser scribed graphene showed a specific capacitance of 276 F/g^26^. Although graphene based supercapacitor electrodes shows good performance, they are limited by specific capacitance of around 300 F/g. To improve specific capacitance its composites and hybrids are being used.

Yanwu Zhu et al. reported a specific capacitance of 166 F/g by using GO^27^. Hsieh et al. used GO in Na_2_SO_4_ and Li_2_SO_4_ and achieved specific capacitance of 98.8 F/g and 238 F/g respectively^28^. Lobato et al. showed that reduced GO showed a specific capacitance of 84 F/g in an H_2_SO_4_ electrolyte^29^. A well-defined composite of Graphene/GO/GrO can achieve both high specific capacitance and energy density. Graphene composites with other materials like oxides, carbon-based materials CNTs etc. have higher capacitive properties. To the best of our knowledge few studies have investigated the effect of GO and GrO in supercapacitors and whereas no study has been reported yet on Graphene/GO/GrO nanocomposite.

In this article, we have studied the effect of sonication time on exfoliation of graphite powder for cost effective and mass scale production of graphene. Three different samples were collected at sonication time of 24 h,48 h and 72 h and characterization techniques such as XRD and RAMAN were used to confirm the exfoliation of graphite powder into graphene, GO and GrO. AFM, SEM and EDS were further employed to observe layered structure of the nanocomposites. UV–Visible Spectroscopy was performed to analyze the effect of sonication on the bandgap of nanocomposite material. I-V, CV, GCD and EIS analysis were also performed to test the electrical and capacitive behavior of the nanocomposite. This article provides a scalable, green, and low-cost method to prepare graphene and its composites for supercapacitor applications.

## Methods

### Materials

Graphite powder (1000 mesh flakes), Polyvinylpyrrolidone (average molecular weight 10,000 DA), Iso-propyl alchohal (Molecular Weight 60.10) and Nickel foam were purchased from Sigma- Aldrich Chemical Co.

### Synthesis of graphene/GO/GrO nanocomposite

Ultrasonic Assisted Liquid Phase Exfoliation of Graphite (UALPE) was used in order to produce graphene of high quality which is economical and scalable. Graphite powder was dispersed in Isopropyl alcohol (IPA) and Polyvinylpyrrolidone (PVP) was added as a stabilizer and this suspension was sonicated in a bath sonicator at different time stamps at 60 °C and atmospheric pressure. After sonication, the graphite layers are isolated by overcoming the van der Waal’s forces which are quite weak and graphene flakes are dispersed in the suspension. For 20 mg of Graphite powder (Sigma-Aldrich, 1000 mesh flakes), 1.5 mg of PVP (Sigma-Aldrich average molecular weight 10,000 DA) were dispersed in 20 ml of IPA. This suspension was then centrifuged at 4030 rpm for 1 h. After centrifugation, the dispersion is settled into two distinct layers, hereafter referred as top and bottom portion (residue). The top layer forms 70% of the dispersion with graphene flakes suspended in IPA and the bottom layer (30%) is residue material. The top 70% suspension is separated. The bottom 30% part is diluted in IPA 5% by volume. More details are given in supplementary information Fig. S1. A series of three experiments were carried out in parallel with sonicator at 60 °C over the intervals of 24 h (sample A), 48 h (sample B), and 72 h (sample C) to study the effect of sonication time on the exfoliation of graphite powder. The heavier residual powder settles down at the bottom of the vial whereas exfoliated flakes suspended in IPA making the top 70% of suspension in the vial. The characterization of top 70% suspension and bottom 30% B was performed. More details on synthesis of Graphene/GO/GrO nanocomposite are given in supplementary information with Supplementary Fig. 1 giving synthesis flow diagram.

### Preparation of electrode for cyclic voltammetery

The electrode fabrication process involved the dissolution of 80% active material, 10% Polyvinylidene fluoride (PVDF) as a binder agent, and 10% carbon black in mass in NMP (n-methyl-2-pyrrolidone) to form a slurry. The slurry was coated on Ni foam and dried in a vacuum oven overnight. The resulting electrode was then pressed at 5 MPa to ensure proper functioning. Supplementary Fig. 2 represent the preparation of electrode process.

### Instrumentation

XRD diffractometer (X-Ray Diffractometer, DRON 8, BRUKER UNITED STATES) was used for the structural analysis of composite having Cu Kα as an X-ray source. Raman shifts were measured using Xplora ONE, Horiba Scientific Raman diffractometer with an exciting laser source of 532 nm. UV–Visible spectrophotometer (UV Visible spectrophotometer, SPECORD 200 plus, Analytik Jena Germany) was used for measuring absorbance spectrum of samples over the range of 100 nm to 350 nm. Scanning Electron Microscopy (SEM) SEM, 6490A, JEOL Japan) and energy dispersive spectroscopy (EDS) were used to study morphology and elemental distribution. SEM was performed at a resolution of 5 µm, 2 µm, 1 µm and 500 nm. Two probes method electrical measurements were performed with sourcemeter (IV measurements B2900 SMU, Keysight). Gamry cyclic voltammeter (Interface 1010E Potentiostat/Galvanostat/ZRA Gamry instruments) was used for electrochemical analysis. Three electrode setup was used for measuring specific capacitance using platinum wire as counter electrode and Ag/AgCl as reference electrode. Bath sonicator (Elma E15h, Elma Electronics, USA) was used for sonication.

## Results and discussions

### Scanning electron microscopy of top 70% suspension

**Figure 1:**
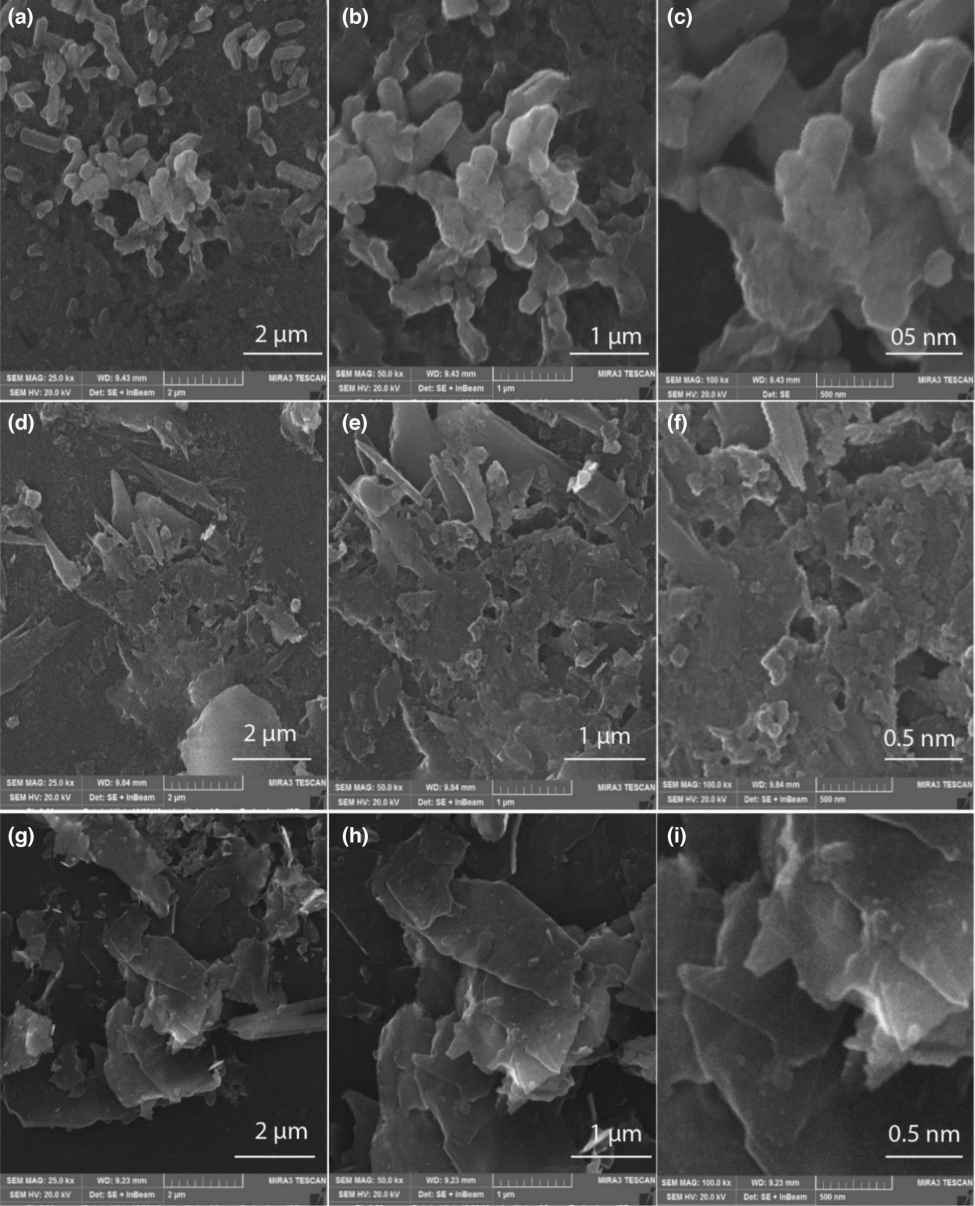
(**a**–**c**) SEM images of sample A, which was sonicated for 24 h produced coagulated particles, (**d**–**f**) SEM micrographs of sample B with a sonication time of 48 h resulted in the formation of coagulated particles and the exfoliated flakes with clear edges (**g**–**i**) SEM images of sample C which was sonicated for 72 h, it has produced isolated layered structure of exfoliated graphene. Scale bars are indicated with each image.

**Figure 2:**
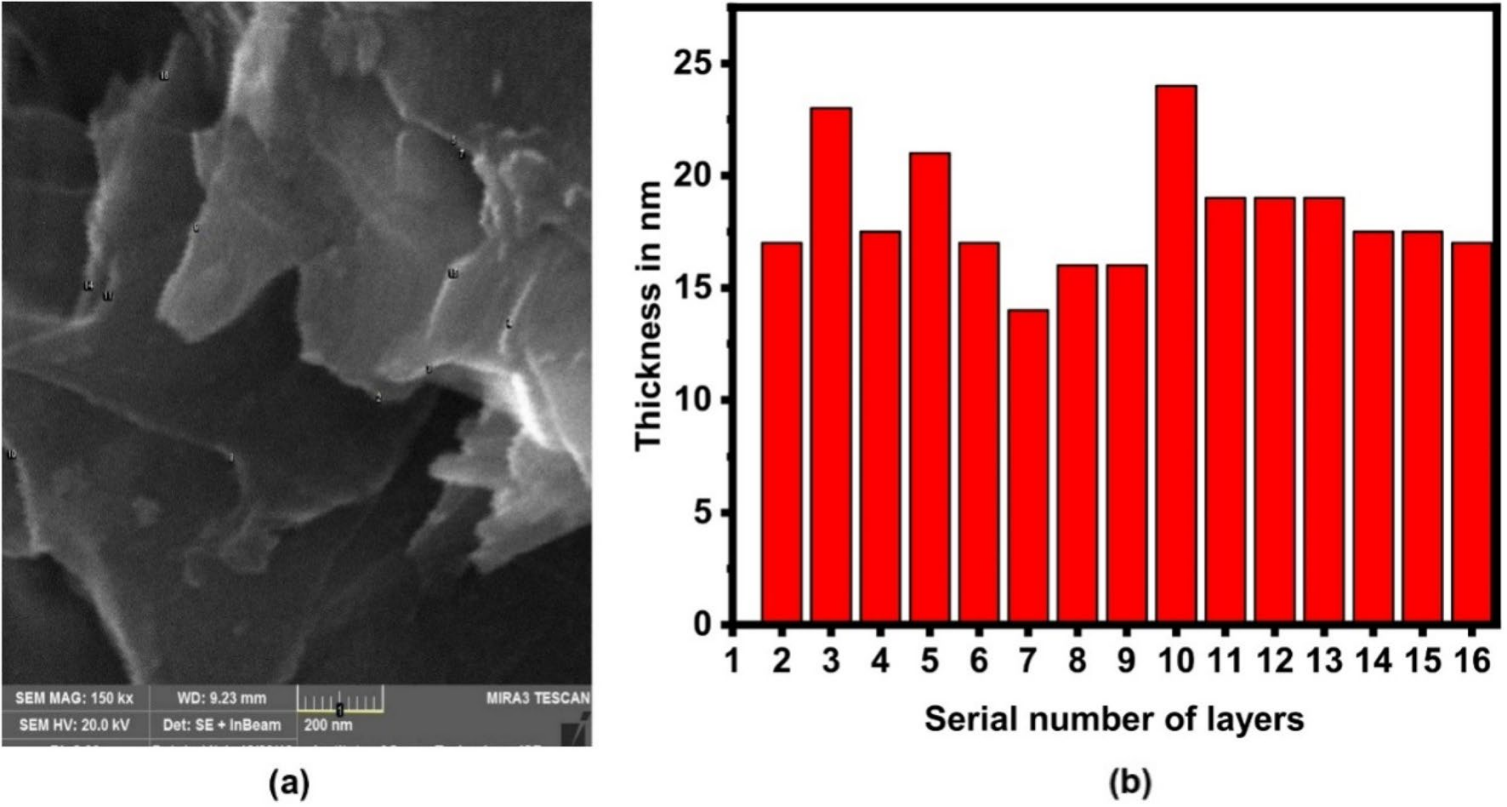
(**a**) SEM image of sample C at 200 nm. (**b**) Bar graph showing thickness profile of different layers in sample C.

Sample A was sonicated for 24 h and its SEM images were taken at 2 μm, 1 μm, and 500 nm. SEM images show coagulated particles as illustrated in Fig. 1a–c. The flakes exfoliated after 24 h of sonication show coagulation instead of any layered structure. Sample B was sonicated for 48 h and its SEM images show coagulated particles with exfoliated flakes having clearly defined edges and layers in the structure as illustrated Fig. 1d–f. In this case, a significant amount of exfoliation has occurred as distinct layers are exfoliated from the chunk of the material. Figure 1g–i, shows the isolated layered structure of exfoliated graphene flakes for sample C. To further investigate the morphology of sample C, it was further analyzed at 200 nm under SEM as illustrated Fig. 2a. SEM results indicated that multilayered graphene of 14.1 nm thickness was achieved. This thickness is calculated using Jpeg software^30^. According to literature, single graphene layer has ~ 0.335 nm thickness and bilayer graphene has 0.81 nm thickness with an interlayer distance of 0.14 nm between two single layer graphene nanosheets^31,32^. Using these values, we calculated that 14.1 nm thick graphene contains 17 layers of carbon atoms i-e 17 monolayer graphene nanosheets. Layers stacked on top of one another with thickness ≥ 100 nm are graphite. In nutshell multi-layered graphene was synthesized as represented in Fig. 2b.

### Atomic force microscopy (AFM) of top 70% suspension

**Figure 3.**
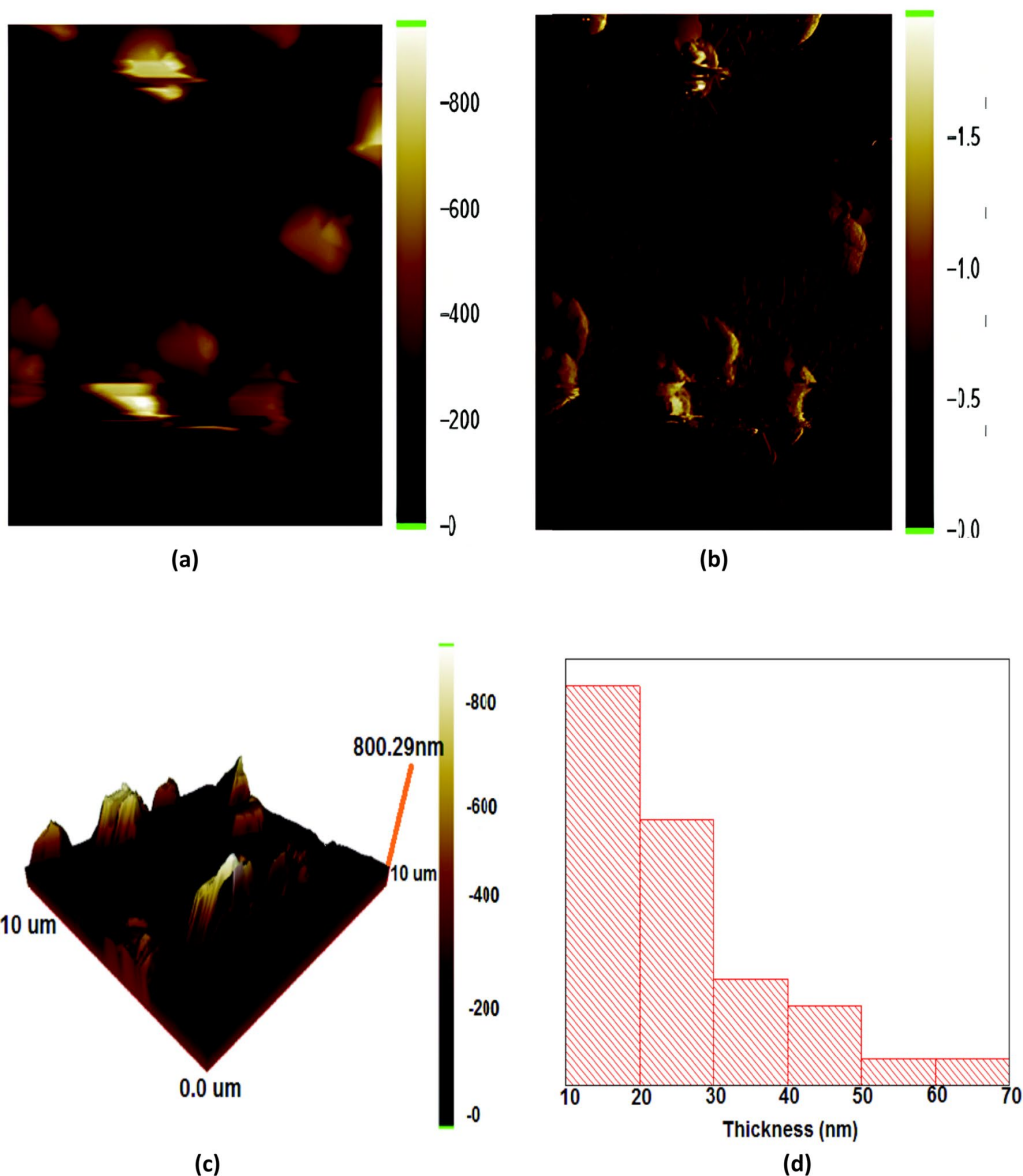
(**a**–**c**) AFM images of sample C. The graphene was grown on glass surface and the sample was scanned in tapping mode with a tapping frequency of 60 kHz, step size of 10 nm. Image size in both (**a**–**c**) are is 10 µm × 10 µm, (**a**) is AFM topography, (**b**) is AFM amplitude and (**c**) is 3D topography representation of (**a**), (**d**) represent Thickness distribution obtained from the AFM image.

AFM probe with a tip of 10–20 nm in diameter was used for AFM scanning. AFM images (Fig. 3a–c) can be used to measure the thickness, roughness, height, and particle size distribution of the specimen. Graphene in sample C, showed promising results in SEM analysis as compared to the other samples. So sample C was further analyzed using AFM. Thickness distribution was obtained from AFM images as shown in Fig. 3d. AFM images revealed that the major area of the sample had a thickness between 10 to 20 nm. This suggests that the graphene flakes in the sample have a thickness within this range, which corroborates the SEM results.

### UV–visible spectroscopy of top 70% suspension

**Figure 4:**
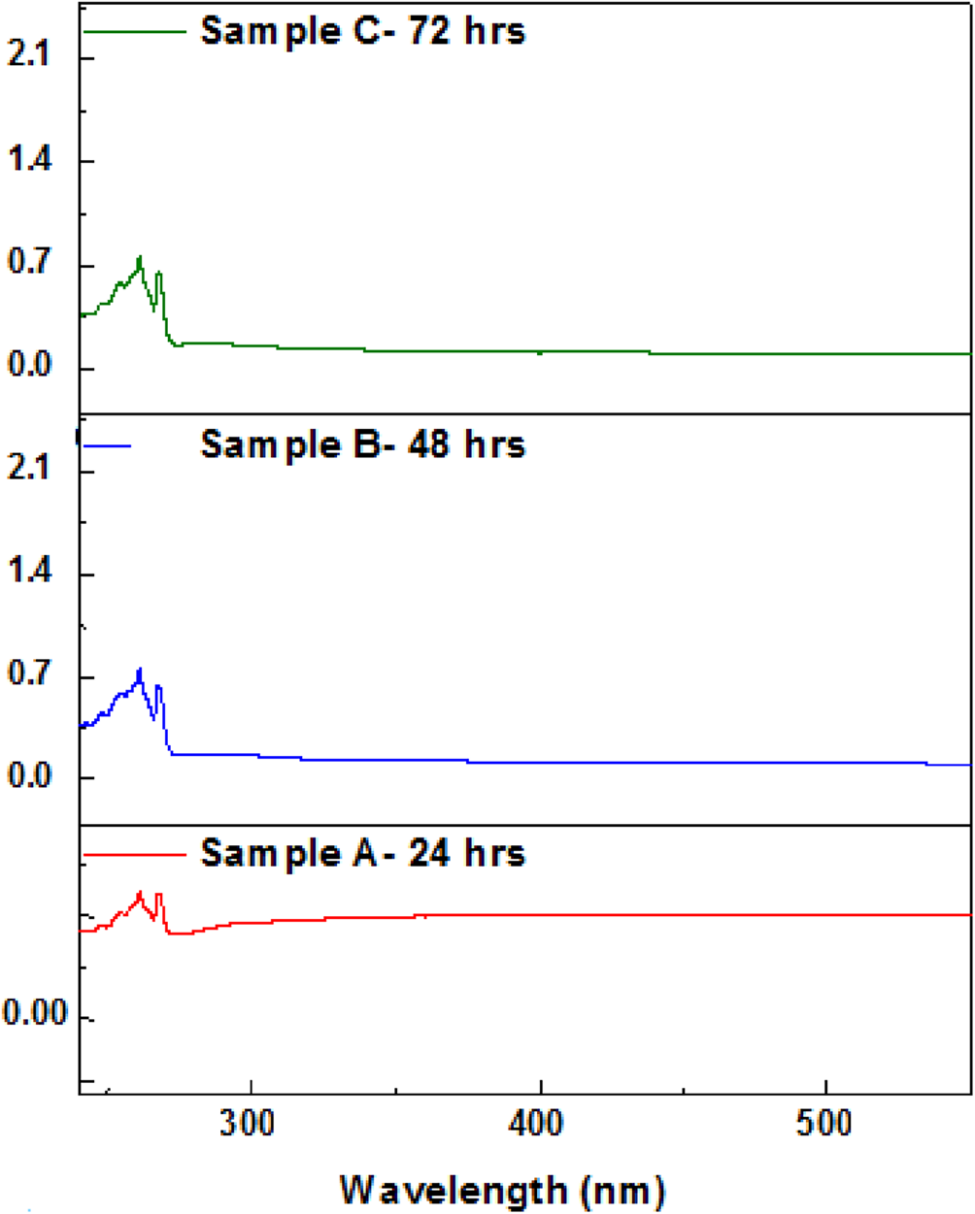
UV spectrum of top 70% suspension for sample A, B and B.

Based on the UV–visible spectroscopy results (Fig. 4), it is observed that all three samples (A, B, and C) show absorbance peaks at around 265 and 270 nm, which are similar to the peaks observed for few layers thick graphene reported in literature^33,34^. This indicates the presence of graphene in the suspensions. These peaks appear due to π–π* transitions. The shift shown in red colored line is observed in all samples which represent change of electronic configuration due to exfoliation of graphite to graphene. The number of graphene layers can be represented by variation in the intensity of peaks^35^. Sample A shows very small absorbance peaks, indicating a low concentration of graphene flakes in the solution. This suggests that there is insignificant exfoliation of graphene from graphite powder in this sample. On the other hand, samples B and C show higher intensity peaks, indicating a higher concentration of exfoliated graphene flakes in the suspension. This suggests a relatively higher degree of exfoliation of graphene from graphite in these samples. Overall, UV–visible spectroscopy results suggest the formation of graphene in the suspensions, which is consistent with the results obtained from SEM and AFM.

Band gap calculation was done on absorption spectrum of all samples by using Tauc Plot method using:

1 $$\alpha hv=A{(hv-{E}_{g})}^{n}$$

where $\alpha$ is the absorption co-efficient, $hv$ is the energy of photon, $A$ is proportionality constant, ${E}_{g}$ represents the optical band gap, and $n$ is the parameter of specific electronic transition within the band due to the light absorption ($n=\frac{1}{2} )$ for direct or 2 for indirect transition. Sample A showed the highest bandgap energy of 5.5 eV, followed by sample B with a bandgap energy of 5.43 eV and sample C with a bandgap energy of 5.22 eV.

This trend was consistent when calculations were performed for the bottom 30% solution. It's worth noting that the top 70% solution was too dilute to perform certain characterizations like XRD and IV due to sensitivity issues with the equipment. As a result, the bottom 30% solution was further analyzed and which is discussed.

### X-ray diffraction of bottom 30%

**Figure 5:**
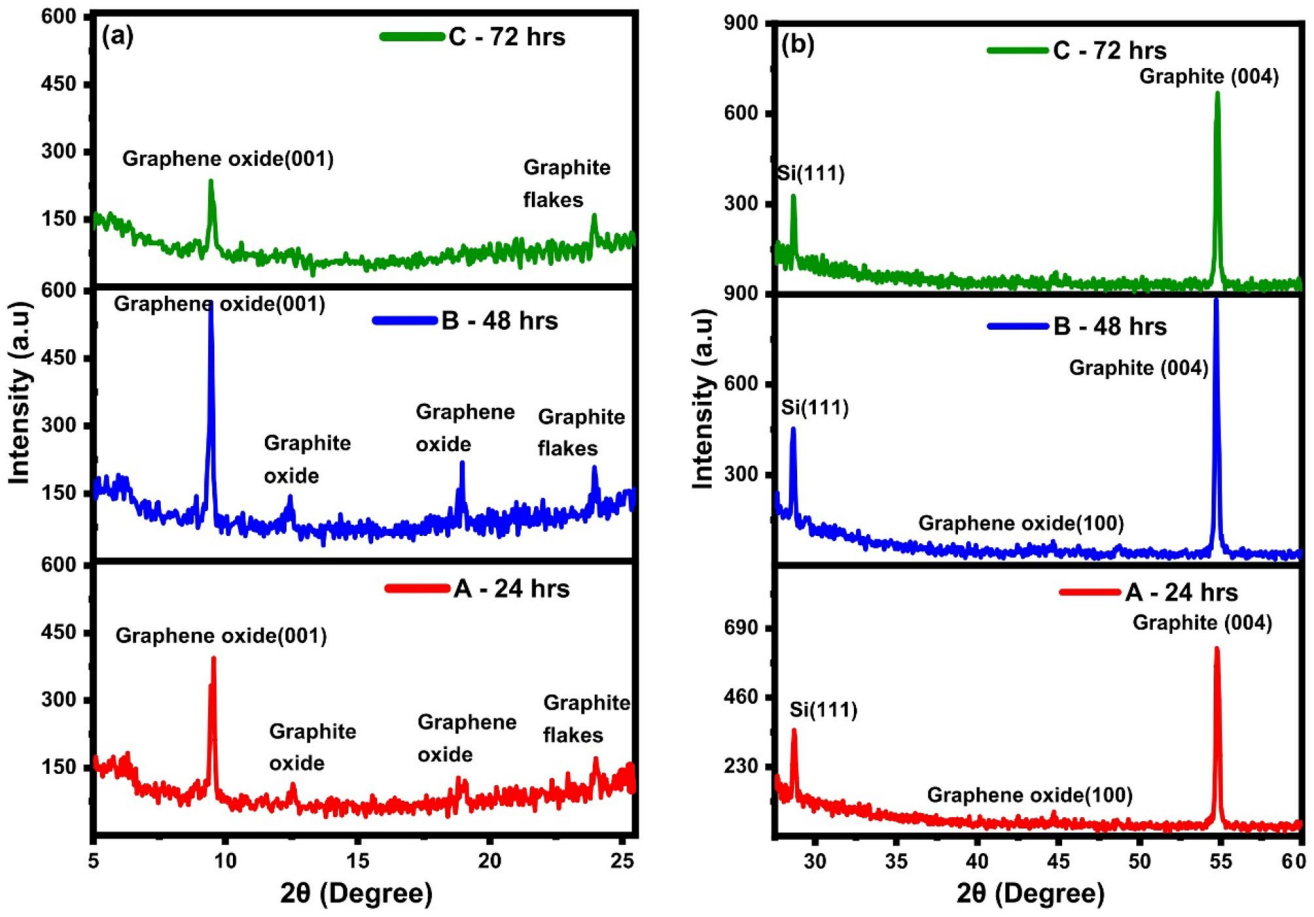
XRD of Sample A, B and C from (**a**) 2$\theta$ = 5$^\circ$ to 25$^\circ$, (**b**) 2$\theta$ = 25$^\circ$ to 60$^\circ$.

The XRD pattern of the samples is shown in Fig. 5, and the most prominent peak in the residual material is the (002) peak at $2\uptheta =26.70^\circ$ of graphite. The peak for sample A was observed to be slightly shifted to $26.65^\circ$, and further shifted to $26.60^\circ$ for sample B and C. The value of d-spacing was calculated using Bragg's law (more details on calculations are given in supplementary information), which relates the angle of diffraction to the wavelength of the X-ray and the distance between the crystal planes. The formula is

2 $$d=\frac{n\uplambda }{2\mathrm{sin\theta }} ,$$

where $d$ represent the d-spacing value, $n$ is the mode of vibration, $\uplambda$ is the wavelength of the X-ray (in this case,$Cu K(\alpha )$ with a wavelength of 1.54 Å), and $\uptheta$ is the angle of diffraction. We use second mode of vibrations for the analysis^36^. The calculated value of d-spacing for sample A is 0.3342 nm corresponding to $2\uptheta =26.65^\circ$, while for sample B and C, this value is calculated to be 0.3348 nm corresponding to $2\uptheta =26.60^\circ$.

The increase in the value of d-spacing in graphite is attributed to the incorporation of oxygen atoms on the basal planes of graphite^37,38^. When new atoms are incorporated or substituted for some of the host atoms, the structure expands or contracts, depending on the newly incorporated atoms. From the X-ray diffraction results, it is observed that the samples contain GO. The (001) peak of GO at $2\uptheta =9.50^\circ$ shifts to $2\uptheta =29.44^\circ$ from sample A to B and is at $2\uptheta =9.44^\circ$ for sample C. This shift of the (001) GO peak to a smaller value is attributed to the increase in d-spacing value due to the incorporation of more oxygen atoms. The increased level of oxidation has increased the d-spacing and expanded the structure slightly. The d-spacing has gone from 0.930 to 0.936 nm from sample A to B, indicating an increase in the interlayer distance between GO sheets. Interplanar distance measured from X-ray diffraction using Bragg's law showed a significant increase from 0.33 nm in graphite to 0.93 nm in GO. This increase due to the attachment of the hydroxyl and epoxy functional groups on the basal plane of honeycomb lattice of graphite during its chemical exfoliation^39^. These functional groups make bonds with the π electrons of the carbon atoms resulting in *sp*^*2*^ to *sp*^*3*^ hybridization of carbon atoms. The degree of oxidation, or the amount of functional groups attached to the basal plane of GO, can be monitored through the interplanar distance, which increases with higher oxidation levels and vice versa. Moreover, the (001) peak in the X-ray diffraction pattern of GO is sensitive to changes in interplanar distance, and its intensity can be used to infer the degree of oxidation of GO. Sample B has the most intense (001) peak, indicating a higher degree of oxidation compared to samples A and C. The decrease in intensity of the (001) peak in sample C suggests a lower degree of oxidation compared to sample B. These partially oxidized flakes have un-oxidized sp^2^ hybridized graphitic domain in them surrounded by sp^3^ hybridized GO^40^. This results in the presence of both GO and graphite in the diffraction spectra of the samples. The crystallinity and arrangement of crystallographic planes in the formed nanocomposite material vary in sample A, B and C. Specifically, the XRD peaks for graphene oxide (19°), and graphite flakes increase in intensity from sample A to sample B, whereas for sample C, the GO and GrO peaks vanish completely while the graphite oxide peak becomes very weak. Additionally, the GO (100) peak also disappears in sample C. The amount of graphene in this nanocomposite is small, which may be why it was not detected in the XRD results. Full scale image of XRD is given in Supplementary Fig. 3.

### Scanning electron microscopy (SEM) of bottom 30%

**Figure 6:**
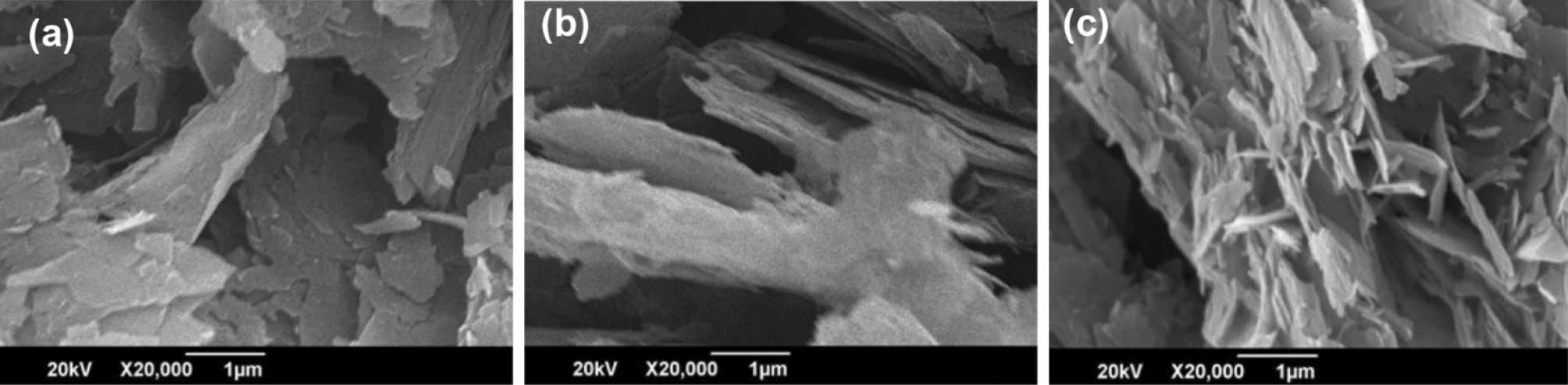
SEM of (**a**) Sample A (**b**) Sample B and (**c**) Sample C.

Scanning Electron Microscope (SEM) was used to examine the structure of three samples taken from the bottom 30% (A, B, and C) at a resolution of 1 µm. Sample A did not show any distinct layered structure and exhibited coagulation which is clear in Fig. 6a. Figure 6b shows SEM image of sample B, which was sonicated for 48 h, exhibited a clear layered structure, although some coagulation was observed. Sample C, which received ample time for sonication, exhibited a very defined layered structure, with separate nanosheets visible in the SEM image shown in Fig. 6c.

### Energy dispersive X-ray spectroscopy (EDX) of bottom 30%

Energy Dispersive X-ray Spectroscopy (EDX) was used to map and analyze the elemental composition of samples A, B, and C. Table 1 shows the elemental composition of the samples, with all three samples showing a higher amount of carbon and oxygen, indicating the successful exfoliation of graphite powder into graphene oxide (GO) and graphite oxide (GrO). Traces of other metals are also present in the samples as impurities in the precursor graphite powder. EDX gives us atomic ratio of elements in a material. Through those values we have carefully calculated the oxygen to carbon (O/C) ratio and it was used to estimate the level of oxidation graphite has gone through during exfoliation process. Sample A has the lowest oxygen to carbon ratio, indicating less oxidation of graphite powder into GO and GrO. Sample B has the highest ratio, indicating maximum conversion to GO. Sample C shows a 98% atomic % carbon and oxygen concentration with only 2% impurity atoms, indicating an even higher oxidation to GO. Overall, the results indicate successful exfoliation of graphite powder into GO and GrO through oxidation. The EDX spectra of all samples had been presented Supplementary Fig. 4.

**Table 1.** EDX of bottom 30% (**a**) Sample A (**b**) B and (**c**) C.

a		b		c	
Element	Weight (%)	Element	Weight (%)	Element	Weight (%)
C K	78.6	C K	82.8	C K	87.9
O K	12.9	O K	11.3	O K	9.1
Mg K	0.9	Mg K	0.8	Mg K	0.7
Al K	0.7	Al K	0.7	Al K	0.4
Si K	1.7	Si K	2.2	Si K	1.1
Fe K	0.9	Ca K	0.8	Fe K	0.9
		Fe K	1.5		

### Raman spectroscopy of bottom 30%

**Figure 7:**
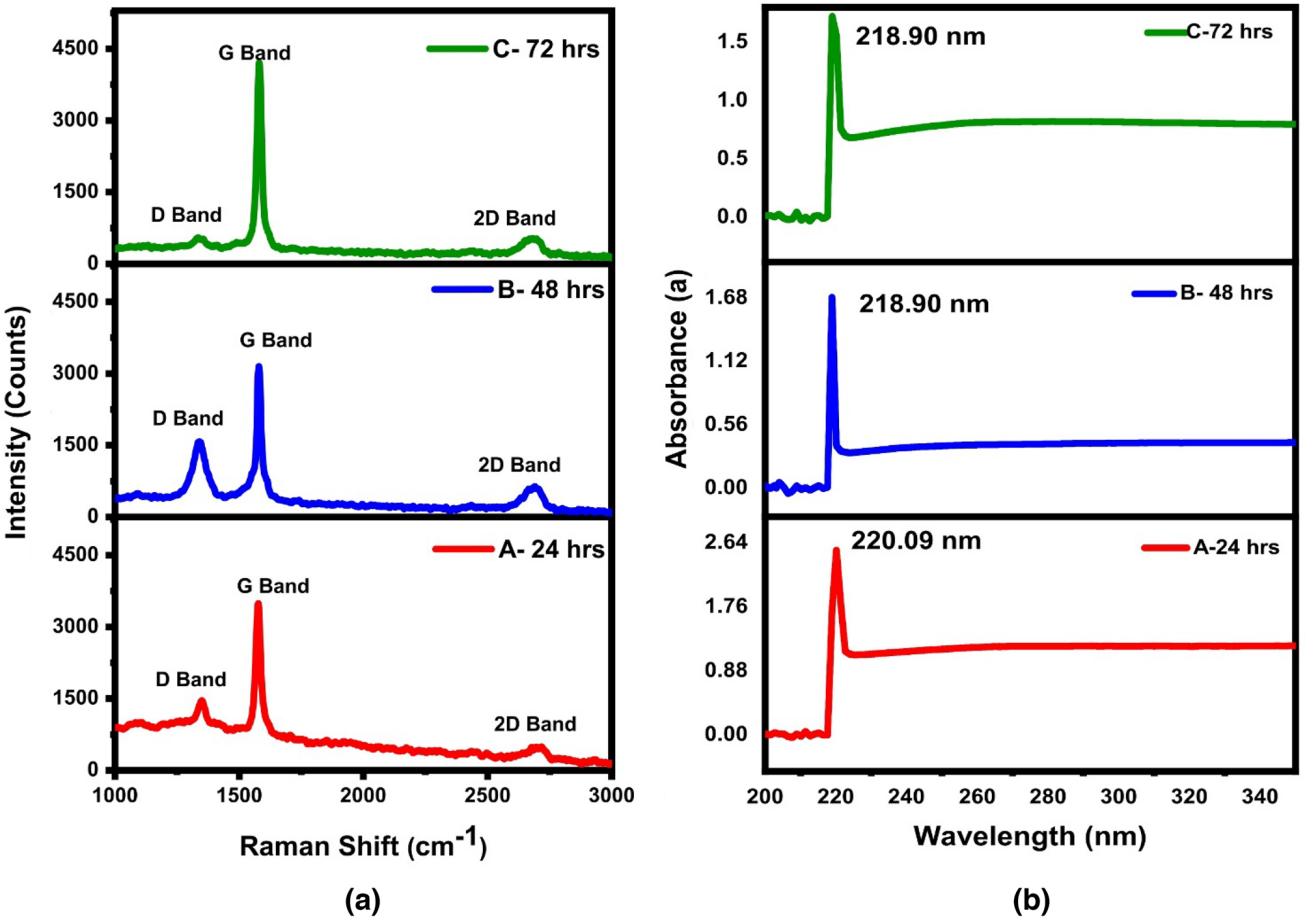
(**a**) Raman Spectroscopy for sample A, B and C, (**b**) UV absorbance spectrum of sample A, B and C.

Raman spectroscopic analysis was performed at room temperature with a laser excitation wavelength of 514 nm to observe the presence of graphene oxide (GO) and the level of oxidation in the samples. Figure 7a shows the Raman spectrum for the samples, with each sample showing the presence of D, G, and 2D bands. The peak at 1366 cm^−1^ corresponds to the D-band, which may be assigned to the A_1g_ mode of vibrations. The formation of the D-band is possibly due to defect-generated phonon mode vibrations or disorders in the honeycomb graphitic structures such as vacancies, bond-angle disorder, edge defects, and bond-length disorder (see supplementary information for more details).

The presence of functional groups like hydroxyl and epoxide on the basal plane of GO nanosheets can also contribute to the occurrence of the D-band^41^. As shown in Fig. 7a, the intensity of the D band increases from sample A to B, indicating an increase in disorder and defects due to increased oxidation level with increase in sonication time. This is consistent with the EDX results ("Energy dispersive X-ray spectroscopy (EDX)of bottom 30%" section), which showed a higher oxygen to carbon ratio in sample B compared to sample A. On the other hand, the decrease in the intensity of the D band from B to C suggests a high defect density regime, where the D band is reduced to a tiny bump due to a highly disordered structure. The width of the D band also increases with an increase in defects and disorder, which is observed from sample A to C due to increased sonication duration. From A to B a decrease in the intensity of the G band is observed as shown in Fig. 7a. This is due to the increased exfoliation effect caused by the removal of oxygen-containing functional groups and the reduction in the number of graphite layers stacked on top of one another. Presence of G and 2D band also affirms our claim of presence of graphene in our nanocomposite^42^. The blue shift of the G band observed from sample A to B suggests an increase in the level of oxidation, which leads to the disruption of graphitic stacked ordering and increased disorder in the material. The increased disorder is reflected in the shift of the G band to a higher frequency, as the phonon frequency of the G band is affected by the degree of sp^2^ hybridization and the degree of disorder in the material. Therefore, the blue shift observed in the G band from A to B indicates the presence of GO or GrO, which have a higher level of disorder and more oxygen-containing functional groups compared to pristine graphene. The increase in the intensity of the G band from B to C is observed because the samples were subjected to coagulation and annealing at an increased temperature and pressure for 72 h. Coagulation can result in the stacking of graphene oxide layers, leading to an increase in the number of graphitic domains, which could increase the intensity of the G band. Additionally, the increased temperature and pressure may have caused further reduction of the graphene oxide, resulting in more ordered graphitic domains and a higher G band intensity.

The I_D_/I_G_ ratio is the ratio of the intensities of the D band and the G band in Raman spectra. This ratio is commonly used to estimate the degree of disorder in graphene-based materials and size of sp^2^ domains in the GO based systems^43^. Different relations like the Knight and White formula^44^ and Tuinstra and Koeng’s relation^45^ can be used to calculate the average sp^2^ domain size in GO based systems, but in our study, we used a general relation by Cancodo et al. to measure the average crystallite size of the sp^2^ domain^46^. These sp^2^ domains gives us information about extent of oxidation of graphite into GO and GrO. This relation gave us a value of

3 $$\frac{{L}_{a}\text{(nm)=}\left(2.4*{10}^{-10}*{\lambda }_{1}^{4}\right)}{\left(\frac{{I}_{D}}{{I}_{G}}\right)}$$

where L_a_ is the average crystallite size of the sp^2^ domains, λ_l_ is the input laser energy, I_D_ is the intensity of the D-band, and I_G_ is the intensity of the G-band. The I_D_/I_G_ ratio increases from A (0.42) to B (0.50) indicating an increase in disorder, number of defects, exfoliation, and oxygen content, which confirms the presence of GO and GrO. This increase in I_D_/I_G_ ratio also reflects a decrease in the size of the in-plane sp^2^ domains and the partially disordered crystal structure of the nanosheets^47^. The decrease in the I_D_/I_G_ ratio from B (0.5) to C (0.13) is due to the highly disordered structure and loss of some crystallinity. The crystallite size is observed to be maximum in sample B, while it decreases in sample A and sample C. This reflects the shrinking of graphitic domains (i.e., disruption of graphitic stacked ordering) and formation of more grain boundaries or lateral defects, which may occur due to expulsion of graphene layers from domains. Therefore, the I_D_/I_G_ ratio can be used to determine the size of sp^2^ domains in GO-based systems.

The I_D_/I_G_ ratio is an indicator of the degree of disorder and the size of sp^2^ domains in GO-based systems. I_D_/I_G_ ratio increases in low defect density regime and decreases in high defect density regime^48^. A higher I_D_/I_G_ ratio corresponds to a greater degree of disorder and a smaller size of sp^2^ domains. Therefore, sample A, with an I_D_/I_G_ ratio of 0.42, has larger sp^2^ domains and is more ordered than sample B, which has an I_D_/I_G_ ratio of 0.50. Similarly, sample C, with an I_D_/I_G_ ratio of 0.13 lies in high defect density regime and has the smallest sp^2^ domains and is the most disordered of the three samples. Overall, the Raman spectroscopic analysis provides further evidence for the presence of GO and GrO as well as the level of oxidation in the samples. Therefore, the Raman spectroscopy results support the EDX findings and provide additional information about the level of disorder and defects in the samples.

### UV–visible spectroscopy of bottom 30%

**Figure 8:**
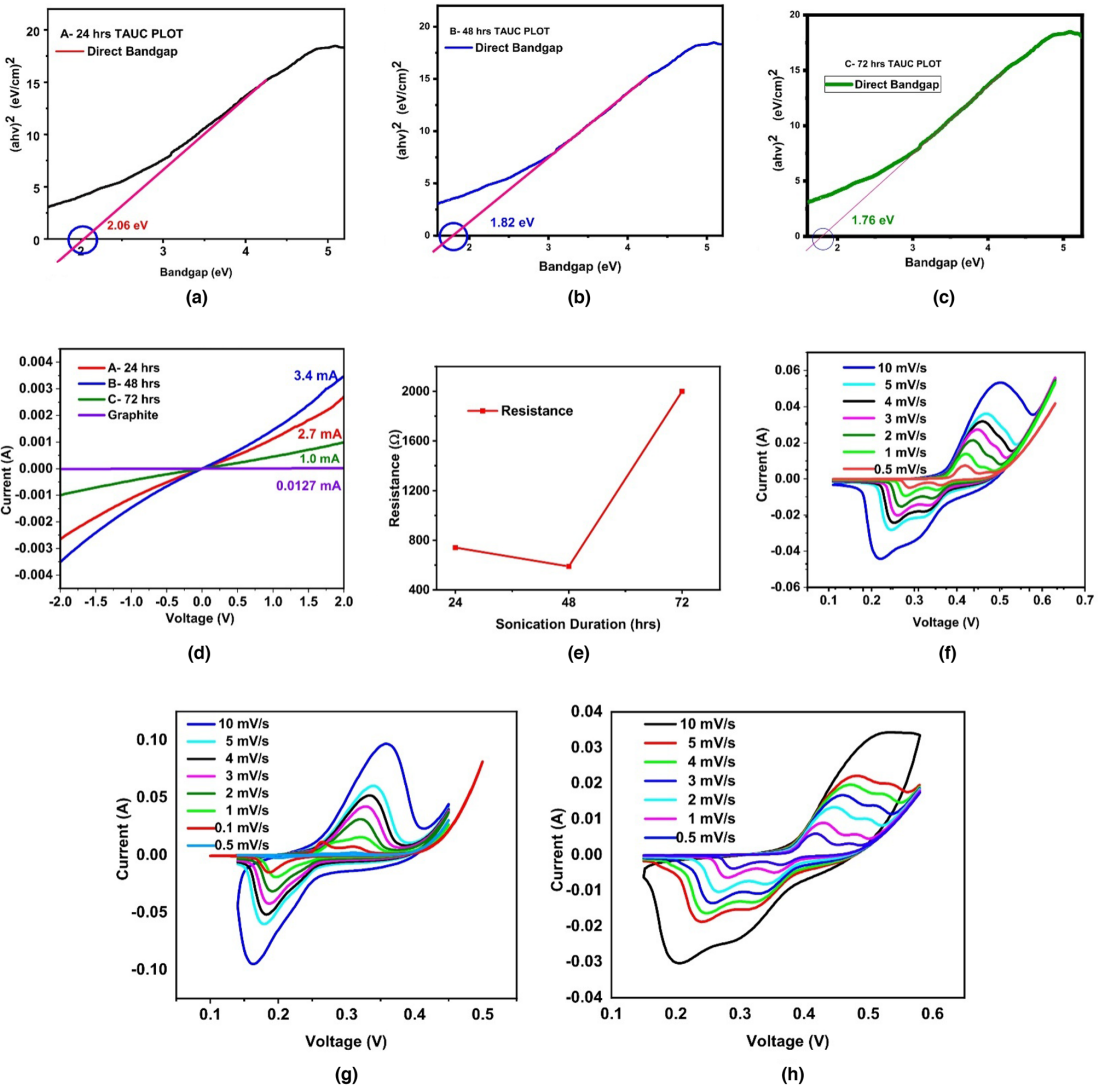
Tauc plot for sample (**a**) A, (**b**) B, (**c**) C, (**d**) I–V analysis for Sample A, B, C and graphite powder, (**e**) resistance graph with respect to sonication duration. (**f**) CV curve of sample A, (**g**) Sample B, (**h**) Sample C at different scan rates.

UV–Visible spectroscopy in the range of 100 nm to 350 nm was conducted to examine the impact of exfoliation on graphite powder. The results indicate that all three samples, A, B, and C, have their maximum absorbance peak at 220.09 nm, 218.90 nm, and 218.90 nm, respectively as shown in Fig. 7b. However, all the samples showed a blue shift. This blue shift suggests that the conjugation in the exfoliated samples was reduced which means that some of graphite is oxidized to form GO and GrO. It was observed that the UV absorbance peak in sample A to sample B was shifted from 220.09 to 218.90 nm, indicating a reduction in conjugation. This reduction in conjugation can be attributed to the oxidation process, which oxidizes some of the graphite to form GO and GrO. The carbon-to-carbon double bonds break to accommodate incoming oxygen and other oxygen-containing functional groups. The shift from 220.09 to 218.90 nm also shows that sample B is more oxidized than sample A. As the level of oxidation increases, the absorbance peak shifts to smaller values of the wavelength. Furthermore, the band gap calculations were performed for all samples on the absorption data by linear extrapolation using the Tauc plot, and the results are plotted in Fig. 8a–c^49^.

The increase in oxidation leads to the formation of the more donor states which in turn can increase the number of conduction states in the material resulting in decreased band gap value. Higher oxygen coverage on surface of graphite causes lattice relaxations which results in the change of band gap^50^. In sample B, with the increase in oxygen content the donor states increase which increases the conduction states as compared to sample A. Other factors like formation of graphite flakes, GrO and GO might also be contributing factors in reduction of band gap. Sample A has the highest bandgap value of 2.06 eV, while sample B has band gap of 1.82 eV and C have 1.76 eV band gap as shown in Fig. 8a–c. The band gap reduces with an increase in sonication time, and this reduction is attributed to the increased oxidation level.

### Electrical properties of bottom 30%

The samples of 5 μm thickness were prepared and subsequently I–V measurements were carried out to observe electrical properties. For I–V measurements, the voltages in the range of − 2 V to 2 V were applied. The I–V curve for each sample was found to be a straight line, indicating Ohmic behavior as shown in Fig. 8d. The I–V curve for the used graphite powder were also analyzed as shown in Fig. 8d. Sample B with the largest slope has the highest conductivity and the smallest resistance, followed by samples A, C and Graphite." The I–V curve for graphite powder had the largest resistance among the samples studied. The resistance of each sample was calculated from the slope of the I–V curve using Ohm's law, which relates the resistance of a material to the slope of its I–V curve. Sample B shows the smallest resistance and highest conductance. For sample B, applied voltage of 2 V produces a current 3.4 mA, which is 2.7 mA, 1.1 mA and 0.0127 mA for sample A, sample C and, graphite powder respectively. Resistance graph in Fig. 8e. shows a minimum resistance of 588 Ω for sample B and maximum resistance of 2 kΩ for sample. Sample A also showed a good conductive behavior which can be attributed to oxidation, increased surface area and formation of GO flakes. Sample B showed the smallest resistance and highest conductance, which indicates that it has the highest conductivity among the three samples. The increased conductivity in sample B can be attributed to the formation of graphene and GrO, which are highly conductive materials, along with the formation of GO flakes and bandgap. This further emphasizes that sample C having least crystalline structure due to more sonication time. XRD, Raman and UV analysis of sample B proves oxidation of graphite to GO. GO itself is not a very good conductor of electricity and is a semiconductor. But in this composite material of sample B, only a very small amount of GO is formed. Just enough to increase the surface area of the material. Along with the formation of Graphene and GrO, the band gap of B is also very small i.e. 1.84 eV. All these factors account for the increased conductivity of sample B. Sample C has the smallest conductivity among these because it has a great amount of disorder and decreased crystallinity which may have resulted in poor carrier mobility. The composite material made in this experiment has highly exfoliated graphite and Graphene oxide (GO) as its main components along with graphene and Graphite oxide (GrO). Presence of GO is not only the reason of Faradic reactions to cause pseudocapacitance but it also increases the surface area of the graphite material. Due to this increase in surface area, an increase in the specific capacitance of the electrodes made by using this composite material is expected which is further tested through Cyclic Voltammetery.

### Cyclic voltammetery measurements of bottom 30%

All samples A, B, and C (bottom 30%) were tested using CV analysis and all of them showed pseudocapacitive behavior, this suggests that these materials have the potential to be used as electrode materials in electrochemical capacitors, such as supercapacitors. Gamry cyclic voltameter with a three-electrode setup was used to measure charge–discharge process, where a platinum wire was used as the counter electrode, Ag/AgCl as the reference electrode, and 1 M KOH as the electrolyte.

Each sample was analyzed at different scan rates ranging between 0.5 and 10 mV/s as shown in Fig. 8f–h. The specific capacitance increases at lower scan rates because potential is swept more slowly, allowing more time for the electrolyte ions to diffuse into the electrode to reach its surface therefore resulting in an increased number of accessible sites, therefore more redox reactions and higher specific capacitance values. Sample A showed the highest specific capacitance value of 205.03 F/g at a scan rate of 1 mV/s. On the other hand, sample B showed a much higher specific capacitance value of 534.53 F/g at a lower scan rate of 0.5 mV/s (Fig. 8g). This high specific capacitance value for sample B can be attributed to its greater exposed surface area, oxidation, and good conductive behavior.

**Figure 9:**
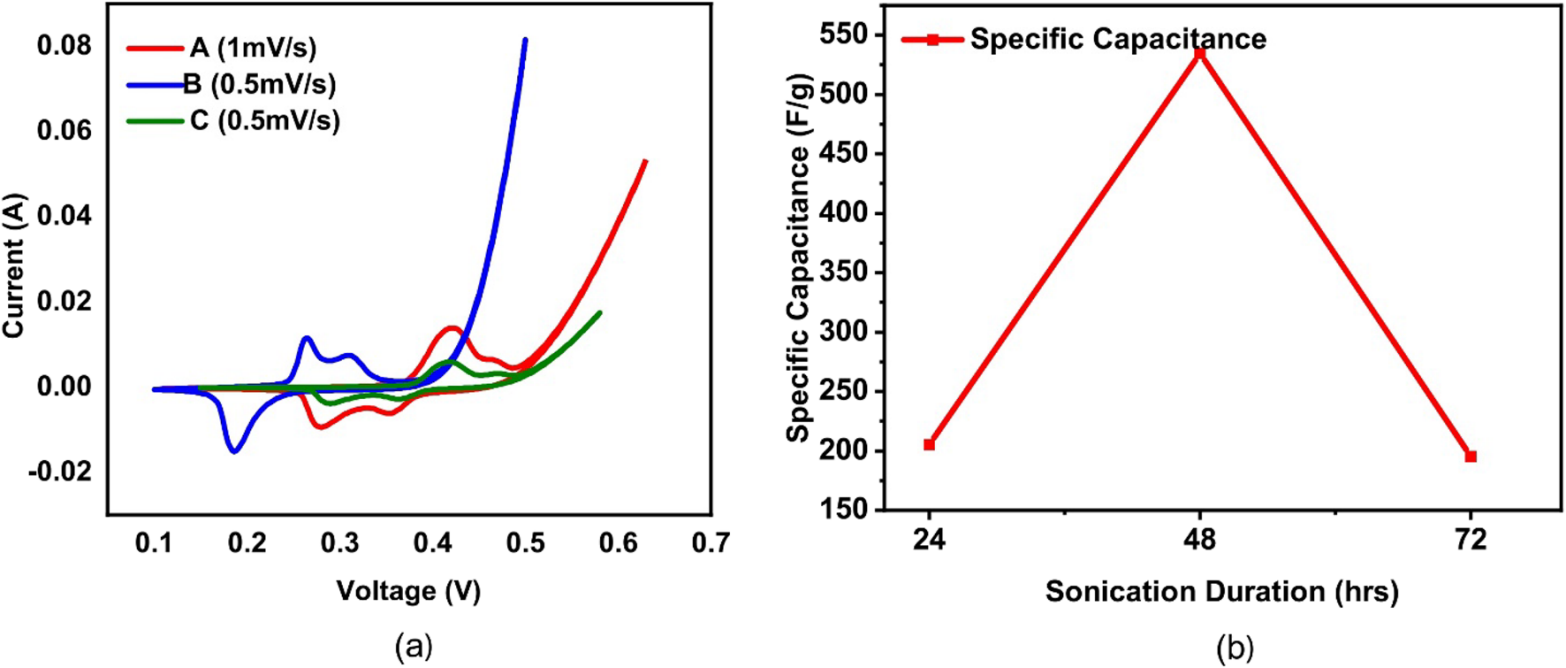
(**a**) CV analysis of sample A, B and C at 1 mV/sec and 0.5 mV/sec. (**b**) Effect of sonication time on specific capacitance.

The presence of oxygen in the form of GO and GrO is the main reason for the pseudocapacitive behavior observed in the nanocomposite material. Sample B shows better results than sample A because it has a higher level of oxidation, which makes it more conductive and hence able to participate in more redox reactions. On the other hand, sample C shows weaker pseudocapacitive behavior, as indicated by the relatively weak redox peaks observed in the CV analysis. Specific capacitance decreases with the increase in scan rate due to increased number of inaccessible sites on the surface of the electrode. Conversely, a lower scan rate enables access to more sites on the surface of the electrode, resulting in a larger specific capacitance value. For sample C, the highest specific capacitance value of 195.12F/g was observed at a scan rate of 0.5 mV/s, as demonstrated in Fig. 8h. As compared to samples A and B, decreased surface area and poor conductive behavior attributed to this relatively low value of specific capacitance for sample C as shown in Fig. 9 (see supplementary information for more details).

Graphite is an insulator in its pure form. Graphene shows excellent electronic properties but its use in energy storage devices is limited because of its weak capacitive behavior due to its limited surface accessibility. Graphene oxide (GO) can enhance its capacitive behavior due to increased sp^3^ hybridization and more oxygen contents, which result in better charge transfer and enhanced Faradaic reactions at the electrode site. When GO is synthesized from graphite, the carbon to carbon double bonds break to incorporate the incoming oxygen and other oxygen-containing functional groups. This process leads to the disruption of graphitic stacked ordering and the formation of more grain boundaries or lateral defects, as observed through Raman analysis.

These defects may occur due to the expulsion of graphene layers from domains, leading to a smaller average size of the graphitic domains. These functional groups form bonds with the π electrons of the carbon atoms thus resulting in hybridization of carbon atoms from sp^2^ to sp^3^. Degree of oxidation or the amount of functional groups attached to the basal plane of GO can be detected by monitoring the interplanar distance. Higher oxidation levels leads toward the increased d-spacing and vice versa. The addition of functional groups to graphene oxide results in more donor sites, which facilitates charge transfer and can lead to improved pseudo capacitive behavior. The increased interlayer spacing in GO compared to graphite and abundant sp^3^ domains are another factors that can contribute to improved pseudo capacitive behavior. This increased spacing provides a better charge transfer network throughout the basal plane, which can enhance conductivity and capacitive behavior. GrO increases the surface area causing more active sites for charge transfer during charging and discharging process. The presence of oxygen in the form of functional groups on GO and GrO is a key factor contributing to the pseudocapacitive behavior in the formed nanocomposite. Furthermore, the lower band gap resulting from oxygen addition can increase the electrical conductivity of the nanocomposite.

Overall, the experiment provides valuable information about the electrochemical properties of composite residual material electrodes made on Nickel foam substrate. The pseudocapacitive behavior exhibited by all samples indicates that these electrodes have promising potential for energy storage applications. The energy density of sample C was found to be lower than that of samples A and B, likely due to its decreased surface area and poorer conductive behavior.

### Galvanstatic charge discharge (GCD)

**Figure 10:**
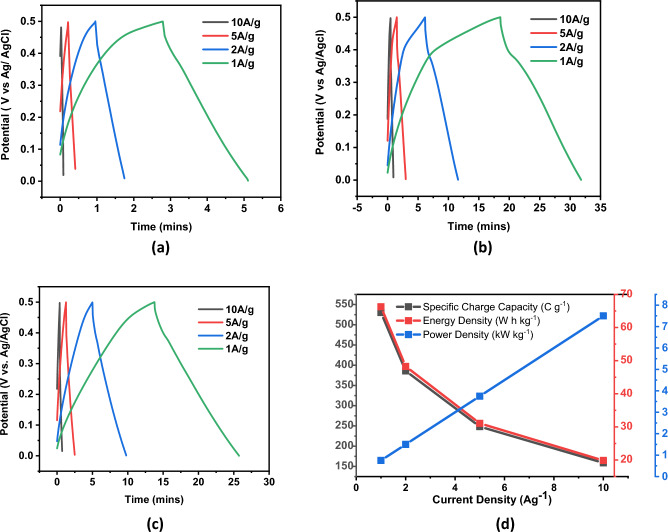
(**a**) GCD plot for Sample A at different current densities, (**b**) GCD plot for Sample B at different current densities, (**c**) GCD plot for Sample C at different current densities, (**d**) Specific charge capacity, Energy density and power density over different current densities for sample B.

Galvanstatic charge discharge (GCD) technique was used to further analyze charge storage mechanism of prepared samples. Nonlinear capacitive GCD curves for sample A, B and C are measured at different current densities of 10 Ag^−1^, 5 Ag^−1^, 2 Ag^−1^ and 1 Ag^−1^ and represented in Fig. 10a–c, respectively. These GCD curves revealed the pseudocapacitive nature of the active material. Specific charge capacity was calculated by $Cp=I\Delta t/(\Delta Vm)$ where $Cp$ is specific charge capacity, I is current density, Δt represents time for charge and discharge cycle, ΔV is voltage difference and m is active mass of electrode material. Energy density and power density is calculated by ${E}_{g}=0.5{C}_{p} {(\Delta V)}^{2}$ and ${P}_{g}={E}_{g}/\Delta t$, respectively. At a current density of 1 A/g, sample A exhibited a charge capacity of 104 c/g. Additionally, it demonstrated an energy density of 8.33 Wh/kg and a power density of 0.6 kW/kg. Sample B exhibited highest charge capacity of 530 c/g at current density of 1 A/g. In addition, the same sample showed transition in power density from 7.5 to 0.75 kW/kg and energy density from 19 to 66 Wh/kg during transition of current density from 10 to 1 A/g. Sample C resulted in a charge capacity of 480 c/g at 1 A/g current density with energy density of 53.60 Wh/kg and power density of 0.7 kW/kg. Figure 10d shows effect of current density on specific charge capacity, energy density and power density for sample B. For cyclic stability, sample B was tested for 5000 cycles at a scan rate of 20 mV/s and it exhibited 94% capacitance retention indicating its ability to maintain a high level of capacitance over time. This characteristic makes it suitable for energy storage applications where long-term stability is important.

### Electrochemical impedance spectroscopy (EIS)

**Figure 11:**
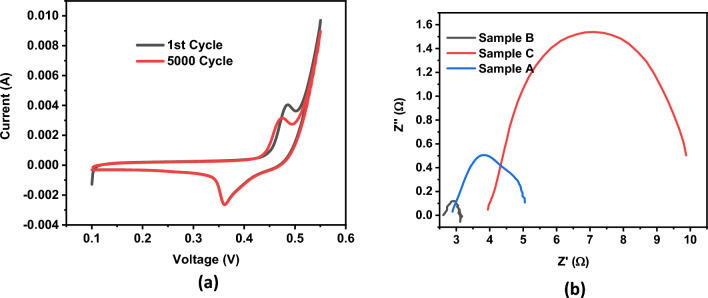
(**a**) Cyclic stability of sample B for 1st and 5000th cycles (**b**) EIS spectrum for all three samples.

Electrochemical impedance spectroscopy (EIS) was employed to measure electrochemical conductivity and analyze the stability and impedance behavior of samples A, B, and C. Figure 11a depicts the stability curves for sample B during the 1st cycle and the 5000th cycle, while Fig. 11b showcases Nyquist impedance plots for all three samples. The presence of semicircles in the Nyquist plots suggests the presence of Faradaic charge storage mechanisms in all samples. Notably, the diameter of the semicircle decreases as the concentration of graphene oxide (GO) increases, indicating improved conductivity and reduced resistance. Comparing the Nyquist plots reveals that sample B exhibits lower resistance compared to samples A and C, signifying enhanced conductivity and greater suitability for electrochemical applications. These findings obtained from EIS analysis provide valuable insights into the electrochemical behavior, stability, and impedance characteristics of the samples, enabling their assessment for various electrochemical applications.

## Conclusion

A cost-effective method for production of Graphene, GO and GrO nanocomposites was developed by tuning sonication time durations for exfoliation. Three graphite powders were sonicated for duration of 24 h, 48 h and 72 h at 60 °C and the resulted nanocomposites were analyzed using AFM, SEM, XRD, Raman, UV–visible spectroscopy and cyclic Voltammetery. The sample B (sonicated for 48 h at 60 °C) showed low resistance and the best pseudocapacitive behavior among the three samples with a specific capacitance of 534.53 F/g specific charge capacity of 530.1 C/g and energy density of 66.2 Wh/kg. Cyclic stability test was performed for over 5000 cycles giving 94% capacitance retention. Morphological and structural characterization suggest that sample B has highly exfoliated graphite and a reasonable amount of graphene, GO and GrO and the conductivity of the Sample B also increased because of presence of exfoliated graphite, GO and GrO. The nanocomposite can have wide spectrum of application in energy storage applications.

### Supplementary Information

Supplementary Information.

## Data Availability

The datasets used and/or analyzed during the current study available from the corresponding author on reasonable request.

## References

[1] Noel Buckley D, O'Dwyer C, Quill N, Lynch RP, Harrison RM, Hester RE (2018). Energy storage options and their environmental impact. Electrochemical Energy Storage.

[2] Sagadevan, S. *et al.* In *Advances in Supercapacitor and Supercapattery* (eds Arshid, M. K. N. & Grace, A. N.) 27–43 (Elsevier, 2021).

[3] Wallace PR (1947). The band theory of graphite. Phys. Rev..

[4] Novoselov KS (2004). Electric field effect in atomically thin carbon films. Science.

[5] Land T (1992). STM investigation of single layer graphite structures produced on Pt (111) by hydrocarbon decomposition. Surf. Sci..

[6] Reina A (2009). Large area, few-layer graphene films on arbitrary substrates by chemical vapor deposition. Nano Lett..

[7] Tang S (2013). Precisely aligned graphene grown on hexagonal boron nitride by catalyst free chemical vapor deposition. Sci. Rep..

[8] Song B (2011). Atomic-scale electron-beam sculpting of near-defect-free graphene nanostructures. Nano Lett..

[9] Singh V (2011). Graphene based materials: Past, present and future. Prog. Mater Sci..

[10] Zhan H, Garrett D, Apollo N (2016). Direct fabrication of 3D graphene on nanoporous anodic alumina by plasma-enhanced chemical vapor deposition. Sci. Rep..

[11] Sellathurai AJ, Mypati S, Kontopoulou M, Barz DPJ (2023). High yields of graphene nanoplatelets by liquid phase exfoliation using graphene oxide as a stabilizer. Chem. Eng. J..

[12] Xie L, Li H (2020). Facile large-scaled fabrication of graphene-like materials by ultrasonic assisted shear exfoliation method for enhanced performance on flexible supercapacitor applications. Appl. Nanosci..

[13] Cai W (2008). Synthesis and solid-state NMR structural characterization of 13C-labeled graphite oxide. Science.

[14] Zhu Y, Murali S, Cai W, Li X, Suk JW, Potts JR, Ruoff RS (2010). Graphene and graphene oxide: Synthesis, properties, and applications. Adv. Mater..

[15] Boehm H-P (2010). Graphene—how a laboratory curiosity suddenly became extremely interesting. Angew. Chem..

[16] Nwamba OC, Echeverria E, McIlroy DN, Austin A, Shreeve JN, Aston DE (2019). Thermal modification of graphite for fast electron transport and increased capacitance. ACS Appl. Nano Mater..

[17] Wang Y, Shi C, Chen Y, Li D, Wu G, Wang C, Guo L, Ma J (2020). Self-supported nickel cobalt carbonate hydroxide nanowires encapsulated cathodically expanded graphite paper for supercapacitor electrodes. J. Electrochim. Acta.

[18] McAllister MJ, Li JL, Adamson DH, Schniepp HC, Abdala AA, Liu J, Herrera-Alonso M, Milius DL, Car R, Prud'homme RK, Aksay IA (2007). Single sheet functionalized graphene by oxidation and thermal expansion of graphite. Chem. Mater..

[19] Nurhafizah MD, Aziz AA, Suriani AB, Mohamed A, Soga T (2019). Low-temperature exfoliated graphene oxide incorporated with different types of natural rubber latex: Electrical and morphological properties and its capacitance performance. Ceram. Int..

[20] Nurhafizah MD, Suriani AB, Mohamed A, Soga T (2020). Effect of voltage applied for graphene oxide/latex nanocomposites produced via electrochemical exfoliation and its application as conductive electrodes. Diamond Relat. Mater..

[21] Mitra S, Sampath S (2004). Electrochemical capacitors based on exfoliated graphite electrodes batteries, fuel cells, and energy conversion. Electrochem. Solid-State Lett..

[22] Liu C, Yu Z, Neff D, Zhamu A, Jang BZ (2010). Graphene-based supercapacitor with an ultrahigh energy density. Nano Lett..

[23] Zhang K, Mao L, Zhang LL, Chan HS, Zhao XS, Wu J (2011). Surfactant-intercalated, chemically reduced graphene oxide for high performance supercapacitor electrodes. J. Mater. Chem..

[24] Kannappan S, Kaliyappan K, Manian RK, Pandian AS, Yang H, Lee YS, Jang JH, Lu W (2013). Graphene based supercapacitors with improved specific capacitance and fast charging time at high current density. Condens. Matter.

[25] Zhu Y, Murali S, Cai W, Li X, Suk JW, Potts JR, Ruoff RS (2010). Ruoff graphene and graphene oxide: synthesis, properties, and applications. Adv. Mater..

[26] El-Kady MF, Strong V, Dubin S, Kaner RB (2012). Laser scribing of high performance and flexible graphene-based electrochemical capacitors. Science.

[27] Zhu Y, Murali S, Stoller MD, Ganesh KJ, Cai W, Ferreira PJ, Pirkle A, Wallace RM, Cychosz KA, Thommes M, Su D, Stach EA, Ruoff RS (2011). Carbon based supercapacitors produced by activation of graphene. Science.

[28] Hsieh CT, Hsu SM, Lin JY, Teng H (2011). Electrochemical capacitors based on graphene oxide sheets using different aqueous electrolytes. J. Phys. Chem. C.

[29] Lobato B, Vretenár V, Kotrusz P, Hulman M, Centeno TA (2015). Reduced graphite oxide in supercapacitor electrodes. J. Colloid Interface Sci..

[30] https://sourceforge.net/projects/jpegview/.

[31] Ni ZH, Wang HM, Kasim J, Fan HM, Yu T, Wu YH, Feng YP, Shen ZX (2007). Graphene thickness determination using reflection and contrast spectroscopy. Nano Lett..

[32] Matei DG, Weber N-E, Kurasch S, Wundrack S, Woszczyna M, Grothe M, Weimann T, Ahlers F, Stosch R, Kaiser U, Turchanin A (2013). Functional single-layer graphene sheets from aromatic monolayers. Adv. Mater..

[33] Güler Ö, Güler SH, Selen V, Albayrak MG, Evin E (2016). Production of graphene layer by liquid-phase exfoliation with low sonication power and sonication time from synthesized expanded graphite. Fullerenes Nanotubes Carbon Nanostruct..

[34] Johra FT, Lee JW, Jung WG (2014). Facile and safe graphene preparation on solution based platform. J. Ind. Eng. Chem..

[35] Uran S, Alhani A, Silva C (2017). Study of ultraviolet-visible light absorbance of exfoliated graphite forms. AIP Adv..

[36] Cullity, B. Elements of X-ray Diffraction. (1978).

[37] Talyzin AV, Sundqvist B, Szabó T, Dmitriev V (2011). Structural breathing of graphite oxide pressurized in basic and acidic solutions. J. Phys. Chem. Lett..

[38] Kim, T. H. *et al.* Enlarging d-spacing of graphite and polarizing its surface charge for driving lithium ions fast. *J. Mater. Chem. A* **2**, 7600–7605 (2014).

[39] Marcano DC, Kosynkin DV, Berlin JM, Sinitskii A, Sun Z, Slesarev A (2010). Improved synthesis of graphene oxide. ACS Nano.

[40] Merazga A, Al-Zahrani J, Al-Baradi A, Omer B, Badawi A, Al-Omairy S (2020). Optical band-gap of reduced graphene oxide/TiO_2_ composite and performance of associated dye-sensitized solar cells. Mater. Sci. Eng. B.

[41] Eda G, Fanchini G, Chhowalla M (2008). Large-area ultrathin films of reduced graphene oxide as a transparent and flexible electronic material. Nat. Nanotechnol..

[42] Sasaki, K.-I. Basic principles of Raman spectroscopy for graphene. *NTT Tech. Rev.* **11** (2013).

[43] Ferrari AC, Robertson J (2000). Interpretation of Raman spectra of disordered and amorphous carbon. Phys. Rev. B.

[44] Knight DS, White WB (1989). Characterization of diamond films by Raman spectroscopy. J. Mater. Res..

[45] Tuinstra F, Koenig JL (1970). Raman spectrum of graphite. J Chem Phys.

[46] Cançado LG, Takai K, Enoki T, Endo M, Kim YA, Mizusaki H (2006). General equation for the determination of the crystallite size L_a_ of nanographite by Raman spectroscopy. Appl. Phys. Lett..

[47] Wang G, Shen X, Yao J, Park J (2009). Graphene nanosheets for enhanced lithium storage in lithium ion batteries. Carbon.

[48] Radoń A, Włodarczyk P, Łukowiec D (2018). Structure, temperature and frequency dependent electrical conductivity of oxidized and reduced electrochemically exfoliated graphite. Phys. E Low-dimens. Syst. Nanostruct..

[49] Viezbicke BD, Patel S, Davis BE, Birnie DP (2015). Evaluation of the Tauc method for optical absorption edge determination: ZnO thin films as a model system. Phys. Status Solidi (b).

[50] Gupta V, Sharma N, Singh U, Arif M, Singh A (2017). Higher oxidation level in graphene oxide. Optik.

