# Supplementary material for: Optimized time dependent exfoliation of graphite for fabrication of Graphene/GO/GrO nanocomposite based pseudo-supercapacitor
Source: Sci Rep. 2023 Aug 30;13:14218. doi: 10.1038/s41598-023-41309-9 (PMC10469176; doi:10.1038/s41598-023-41309-9)
Supplement: Supplementary file 1 — Supplementary Information. [file 41598_2023_41309_MOESM1_ESM.pdf]

Supplementary Information

Optimized time dependent exfoliation of graphite for fabrication of Graphene/GO/GrO nanocomposite based pseudo-supercapacitor

Sana Zainab[1], Sajal Fraz[1], Saif Ullah Awan[1]*, Danish Hussain[2]*, Syed Rizwan[3], Waqar Mehmood[4]

1.Department of Electrical Engineering, NUST College of Electrical and Mechanical Engineering, National University of Sciences and Technology (NUST), Islamabad 54000, Pakistan

2.Department of Mechatronics Engineering, NUST College of Electrical and Mechanical Engineering, National University of Sciences and Technology (NUST), Islamabad 54000, Pakistan.

3.Physics Characterization and Simulations Lab, School of Natural Sciences (SNS), National University of Sciences and Technology (NUST), Islamabad 54000, Pakistan

4.Material Synthesis & Characterizations (MSC) Laboratory, Department of Physics, Fatima Jinnah Women University (FJWU), The Mall Rawalpindi 46000, Pakistan.

✉Corresponding authors.*saifullahawan@ceme.nust.edu.pk,*danish.hussain@ceme.nust.edu.pk,

Experimental Section

Synthesis Procedure

In order to produce graphene with the best quality and economical and scalable method, Ultrasonic Assisted Liquid Phase Exfoliation of Graphite (UALPE) is selected to be the method of choice.Isopropyl alcohol (IPA) is chosen as solvent for dispersion due to its low surface tension(γ=21.79mN/m) and lower boiling point (82.6 °C)[1]. IPA has Hansen solubility parameter mismatch with Graphene and hence cannot be used in its pure form. Polyvinylpyrrolidone (PVP) is highly favored to be used as a stabilizer for graphite / graphene dispersion in IPA[2].Graphite powder is dispersed in Isopropyl alcohol (IPA) and Polyvinylpyrrolidone (PVP) is added as a stabilizer and this suspension is sonicated in a bath sonicator for different durations at 60 □and atmospheric pressure. After sonication, the graphite layers are isolated by overcoming the van der Waal's forces which are quite weak and graphene flakes are dispersed in the suspension[3]. The general scheme of the experiment is explained here. For 20 mg of Graphite powder, 1.5 mg of PVP was used and they were dispersed in 20 ml of IPA. This suspension was then centrifuged at 4030 rpm for 1 hour. After centrifugation, the dispersion is split into two parts. The top layer (70%) is graphene flakes suspended in IPA and the bottom layer (30%) is residue material. The top 70% suspension is decanted off. The bottom 30% part is diluted in IPA 5% by volume. This process is shown in Fig. S1. A series of 3 experiments was carried out in parallel with sonicator at 60 □ over the intervals of 24 hours(sample A), 48 hours(sample B), and 72 hours(sample C). The reason for employing different sonication intervals is to see the effect of sonication time on the exfoliation of graphite powder. The heavier residual powder settles down at the bottom of the vial whereas exfoliated flakes suspended in IPA formed the top 70% of suspension in the vial. In this article, we took this bottom 30% part and observed its properties for supercapacitor applications in of result. Top 70% part of suspension was discussed using SEM, AFM and UV-Visible spectroscopy characterizations which affirmed presence of graphene, GO and GrO in sample but it was observed that its electrical properties measurements are not feasible due to its extremely diluted form.

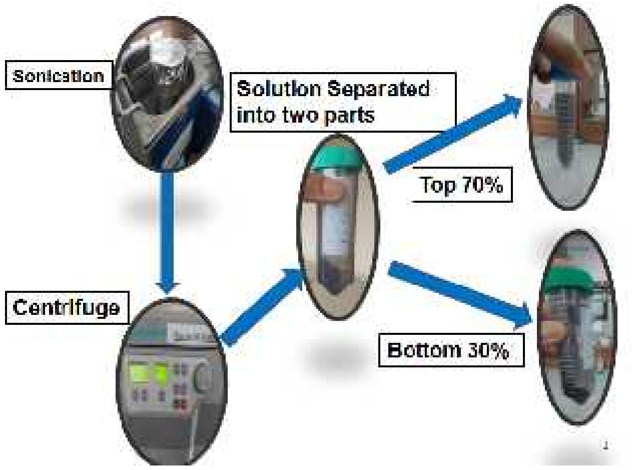

Supplementary Figure 1: Methodology of synthesis of Graphene/GO/rGO nanocomposite using Ultrasonic assisted liquid phase exfoliation.

Electrode fabrication for Cyclic Voltametery

Electrode for cyclic voltametry is made on Nickle foam. Slurry is made in ratio of 80% active material-10% Polyvinylidene fluoride (PVDF) as a binder agent and 10% carbon black in mass. NMP (n-methyl-2-pyrrolidone) is used as a solvent to make slurry and then slurry is coated on Nickle foam and dried in vacuum oven overnight. Ni foam of area 1x1 cm was used to make electrodes to be used for CV analysis. Ni foam was first washed with DI water, ethanol and acetone for 5min each for removing any impurity. After washing it is dried and then used for electrode formation. Using a dropper, the three slurries A, B and C were drop casted onto the Ni foam. 2 mg of suspension i.e. the composite material were deposited on each Ni foam electrode. To make it as working electrode, sample is then pressed at 5MPa. Process is shown in Supplementary figure 2.

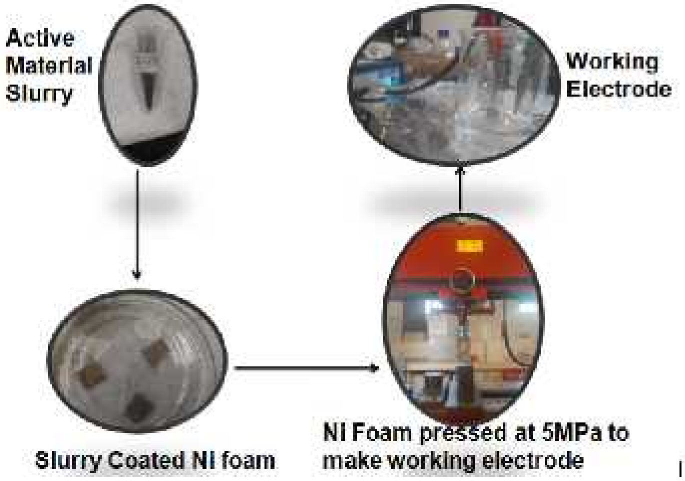

Supplementary Figure 2: Electrode Formation Process

Crystallite size calculations:

Crystallite size (D) is calculated using Debye-Scherrer's eq.

$$\boldsymbol{D} = \frac{\lambda K}{\beta \cos\theta} \text{------------------ (1)}$$ [4].

For (002) peak of graphite in sample A, D is calculated to be 4.3198 Å and for sample B it is 4.2670 Å. A decrease in the crystallite size is observed. This verifies that as crystallite size increase the width of the peak increases[5]. For sample C, the crystallite size is calculated to be D=4.5741 Å. The value of D increases from B to C whereas the FWHM increases. This tells us that with the increase in crystallite size peak width decreases and it gets sharper.There is a prominent loss of crystallinity from sample B to C. This is also indicated by the tip broadening of (002) peak in sample C.

Peaks are observed at the following values of 2θ in XRD of sample A as shown in Fig. 5 of main article with the corresponding values of d-spacing calculated:

9.50°- Graphene Oxide (001), d= 9.30 Å
12.55°- Graphite Oxide d= 7.04 Å
19.05°- Graphene Oxide
24.00°- Graphite flakes
28.70°- Si (111)
44.69°-Graphene Oxide (004) d= 2.02 Å
55.74°- Graphite (004) d= 1.64 Å

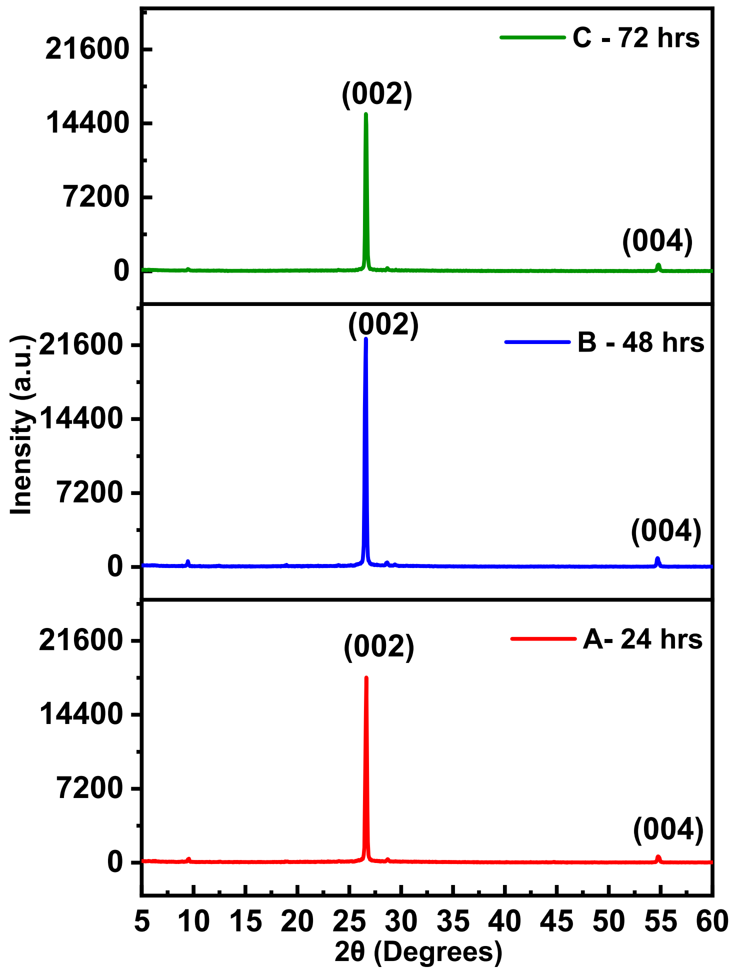

Supplementary Figure 3: XRD spectrum for Sample A, B and C taken from 2θ=5° to 60°.

Peaks are observed at the following values of 2θ in XRD of B with the corresponding values of d-spacing calculated:

9.44°- Graphene Oxide (002), d= 9.36 Å

12.45°- Graphite Oxide, d= 7.05 Å

18.95°- Graphene Oxide

23.95°- Carbon Black

28.65°- Si(111)

44.65°- Graphene Oxide (004), d= 2.02 Å

55.70°- Graphite Oxide (004),   d= 1.64 Å

Peaks are observed at the following values of 2θ in XRD of C:

9.44°- Graphene Oxide (002) , d= 9.36 Å

23.95°- Carbon Black

28.65°- Si(111)

55.80°- Graphite Oxide (004),   d= 1.64 Å

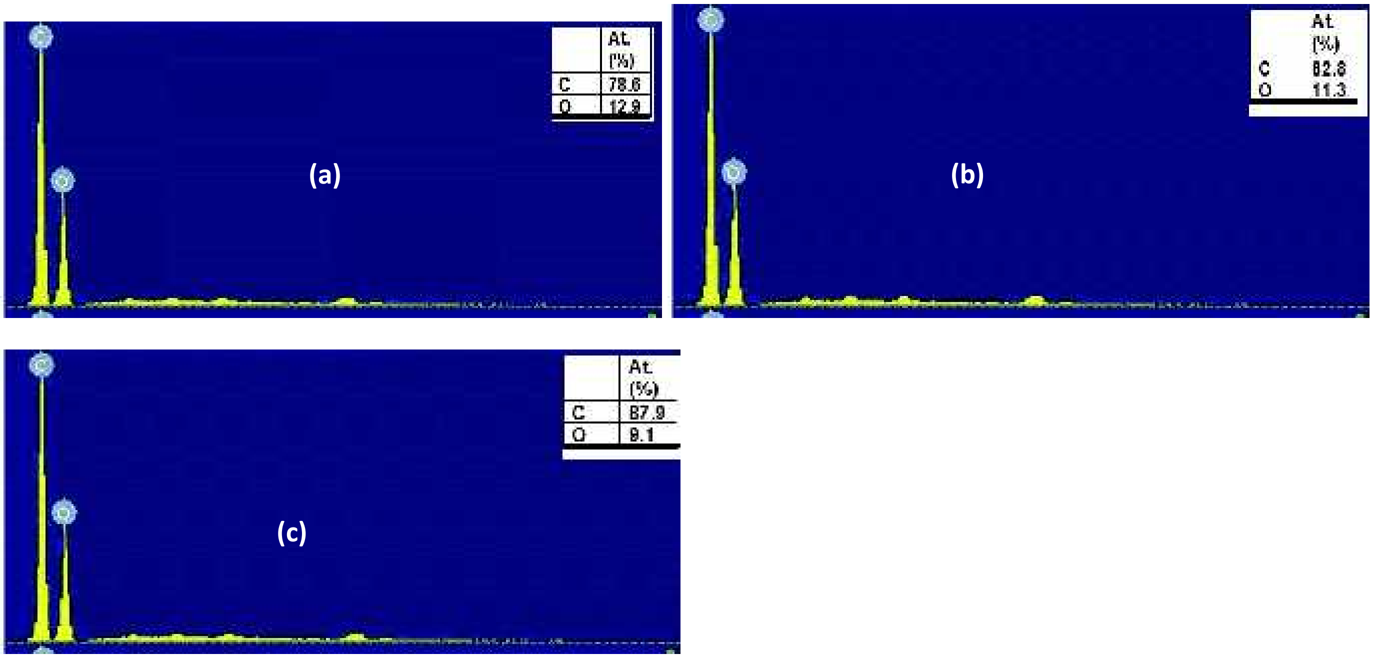

Supplementary Figure 4: EDS spectra for (a) Sample A, (b) Sample B and (c) Sample C.

Position of G band in Raman Spectroscopy

The change in position of G Band to lower wave number (cm^{-1} value on x-axis) is called red shift and shift to the higher wave number is called blue shift. A blue shift of the G band is because of increased oxidation levels and increased defects[6]. From sample A to B the G band exhibits blue shift. This blue shift is because of the increased level of oxidation and because of the increased exfoliation effect and increased disorder[7]. The values of wave numbers for each sample are shown in the table below.

Supplementary Table 1

Sample	G Band Position (cm^{-1})
A	1576.70
B	1580.09
C	1580.09

Supplementary Table 2: Specific Charge Capacity, Energy density and Power Density of all three samples at 1 Ag^{-1}.

	Specific charge capacity (Cg^{-1})	Energy Density ($Whkg^{-1}$)	Power Density ($kWkg^{-1}$)
Sample A	104.1	8.3	0.6
Sample B	530.1	66.2	0.75
Sample C	428.8	53.6	0.72

References

1 S. Haar, e. a. Enhancing the liquid-phase exfoliation of graphene in organic solvents upon addition of n-octylbenzene. *Scientific reports* **5** (2015).

2 Hasan, T., et al. Stabilization and "debundling" of single-wall carbon nanotube dispersions in N-methyl-2-pyrrolidone (NMP) by polyvinylpyrrolidone (PVP) . *The Journal of Physical Chemistry C* **34**, 12594-12602. (2007).

3 S. Haar, et. al. Enhancing the liquid-phase exfoliation of graphene in organic solvents upon addition of n-octylbenzene. *Scientific reports,* **5** (2015).

4 Sharma, N., et al. . Synthesis and characterization of graphene oxide (GO) and reduced graphene oxide (rGO) for gas sensing application. *Macromolecular Symposia* (2017).

5 Guimont, A., et al. Grafting of polyethylene onto graphite oxide sheets: a comparison of two routes. . *Polymer Chemistry* **4**, 2828-2836 (2013).

6 Sharma, N. *et al.* in *Macromolecular Symposia.* 1700006 (Wiley Online Library).

7 Muzyka, R., Drewniak, S., Pustelny, T., Sajdak, M. & Drewniak, Ł. Characterization of Graphite Oxide and Reduced Graphene Oxide Obtained from Different Graphite Precursors and Oxidized by Different Methods Using Raman Spectroscopy Statistical Analysis. *Materials* **14**, 769 (2021).
